# Supplementary material for: CD74 is a regulator of hematopoietic stem cell maintenance
Source: PLoS Biol. 2021 Mar 4;19(3):e3001121. doi: 10.1371/journal.pbio.3001121 (PMC7963458; doi:10.1371/journal.pbio.3001121)
Supplement: S1 Fig — (A–D) Gating strategy of HSPCs. (E) Sorted CD34-/LSK cells were analyzed for CD74 mRNA levels in WT and CD74−/− mice; n = 3. Bars show the DESeq2 normalized counts for the CD74 gene, Data A in S9 Data. (F) Lin- populations from WT and CD74−/− mice were analyzed for cKit and Sca-1 expression by FACS; n = 15, Data B in S9 Data. (G) C57BL/6 and CD74−/− mice were crossed to obtain WT and CD74−/− littermate mice. Percent of CD34-/LSK from WT and CD74−/− littermates; n = 3, Data C in S9 Data. (H) Sorted CD34-/LSK cells were analyzed for MIF mRNA levels in WT and CD74−/− mice; n = 3. Bars show the DESeq2 normalized counts for the MIF gene, Data D in S9 Data. (I) CD34-/LSK population from WT and CD74−/− mice was analyzed for MIF intracellular expression by FACS; n = 13, Data E in S9 Data. (J, K) Sorted CD34-/LSK cells were analyzed for CD44 mRNA levels in WT and CD74−/− mice; n = 3. Bars show the DESeq2 normalized counts for the CD44 gene, Data F in S9 Data (J) and cell surface CD44 expression; n = 6 (K), Data G in S9 Data. Results are presented as mean −+ SD (unpaired two-tailed t test *<0.05 **). The fcs files and gates can be found in FR-FCM-Z3F2. FACS, fluorescence-activated cell sorting; HSPC, hematopoietic stem and progenitor cell; MIF, migration inhibitory factor; WT, wild-type. (PPTX) [file pbio.3001121.s001.pptx]

## Slide 1
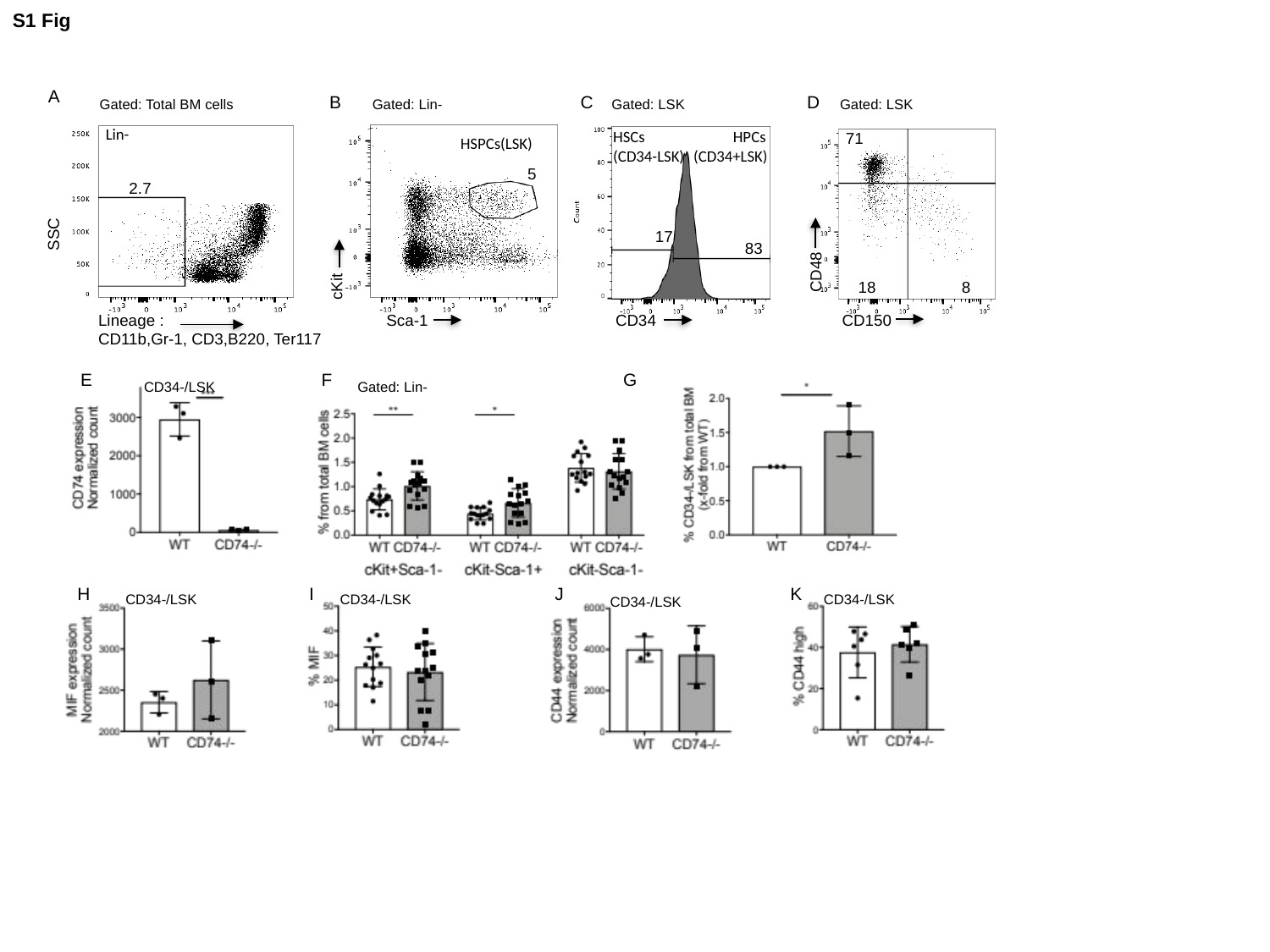

S1 Fig
A
B
C
D
Gated: Total BM cells
Gated: Lin-
Gated: LSK
Gated: LSK
Lin-
HSCs
(CD34-LSK)
 HPCs
(CD34+LSK)
71
HSPCs(LSK)
5
2.7
SSC
17
83
CD48
cKit
18
8
Lineage :
CD11b,Gr-1, CD3,B220, Ter117
Sca-1
CD34
CD150
E
F
G
CD34-/LSK
Gated: Lin-
H
I
J
K
CD34-/LSK
CD34-/LSK
CD34-/LSK
CD34-/LSK
