# Supplementary figures and images for: CD74 is a regulator of hematopoietic stem cell maintenance
Source: PLoS Biol. 2021 Mar 4;19(3):e3001121. doi: 10.1371/journal.pbio.3001121 (PMC7963458; doi:10.1371/journal.pbio.3001121)

## Slide 1
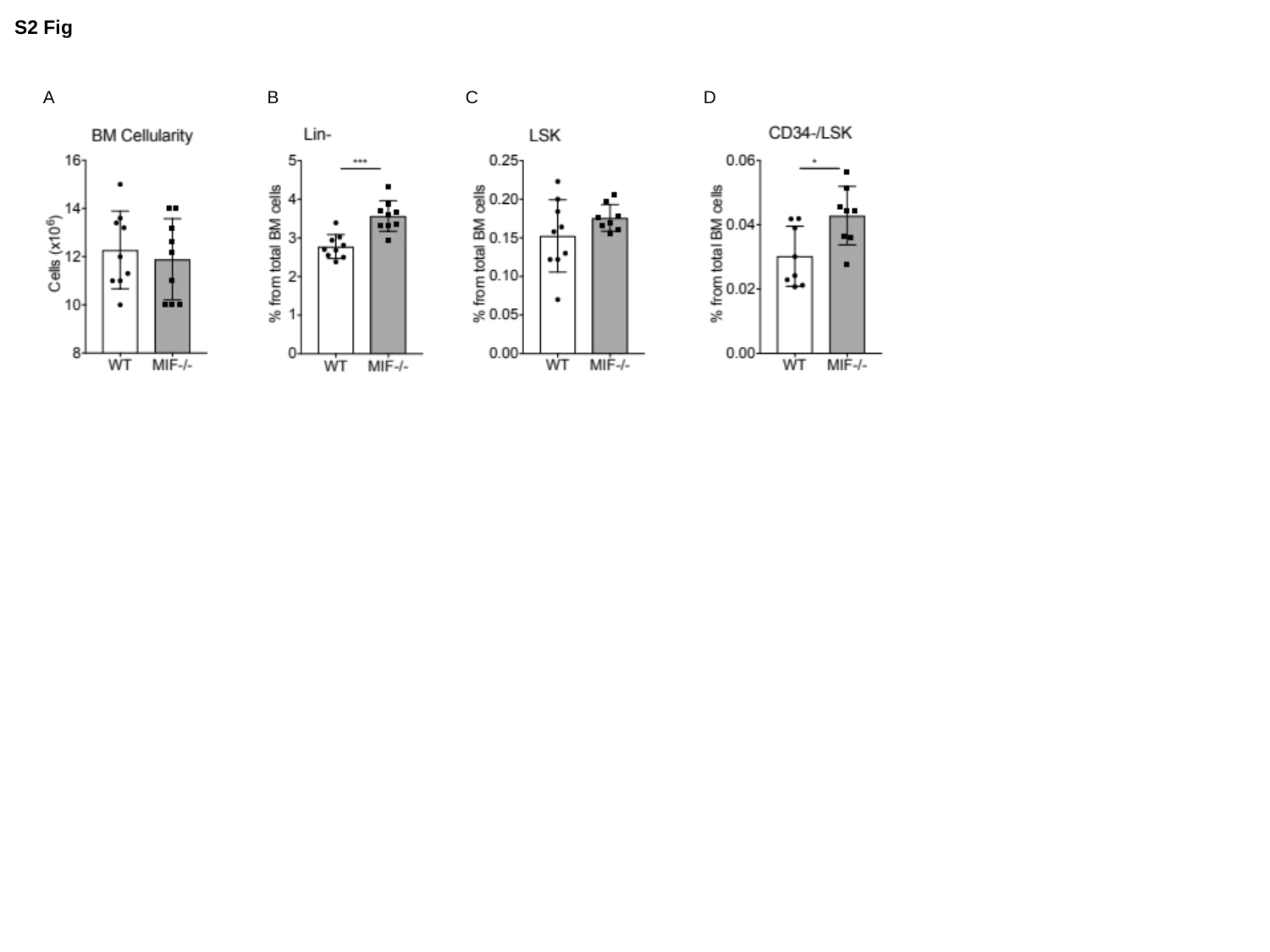

S2 Fig
A
B
C
D

Supplement: S2 Fig — BM cells derived from WT or MIF−/− were purified. (A) Total BM cellularity per femur and tibia, Data A in S10 Data (B–D) The percent of the different populations in WT and MIF−/−-derived BM cells. (B) Lin-; Data B in S10 Data (C) LSK; Data C in S10 Data (D) CD34-; Data D in S10 Data n = 8–9. Results are presented as mean −+ SD (unpaired two-tailed t test *<0.05 **<0.005 ***<0.0005). BM, bone marrow; HSPC, hematopoietic stem and progenitor cell; MIF, migration inhibitory factor; WT, wild-type. (PPTX) [file pbio.3001121.s002.pptx]

## Slide 1
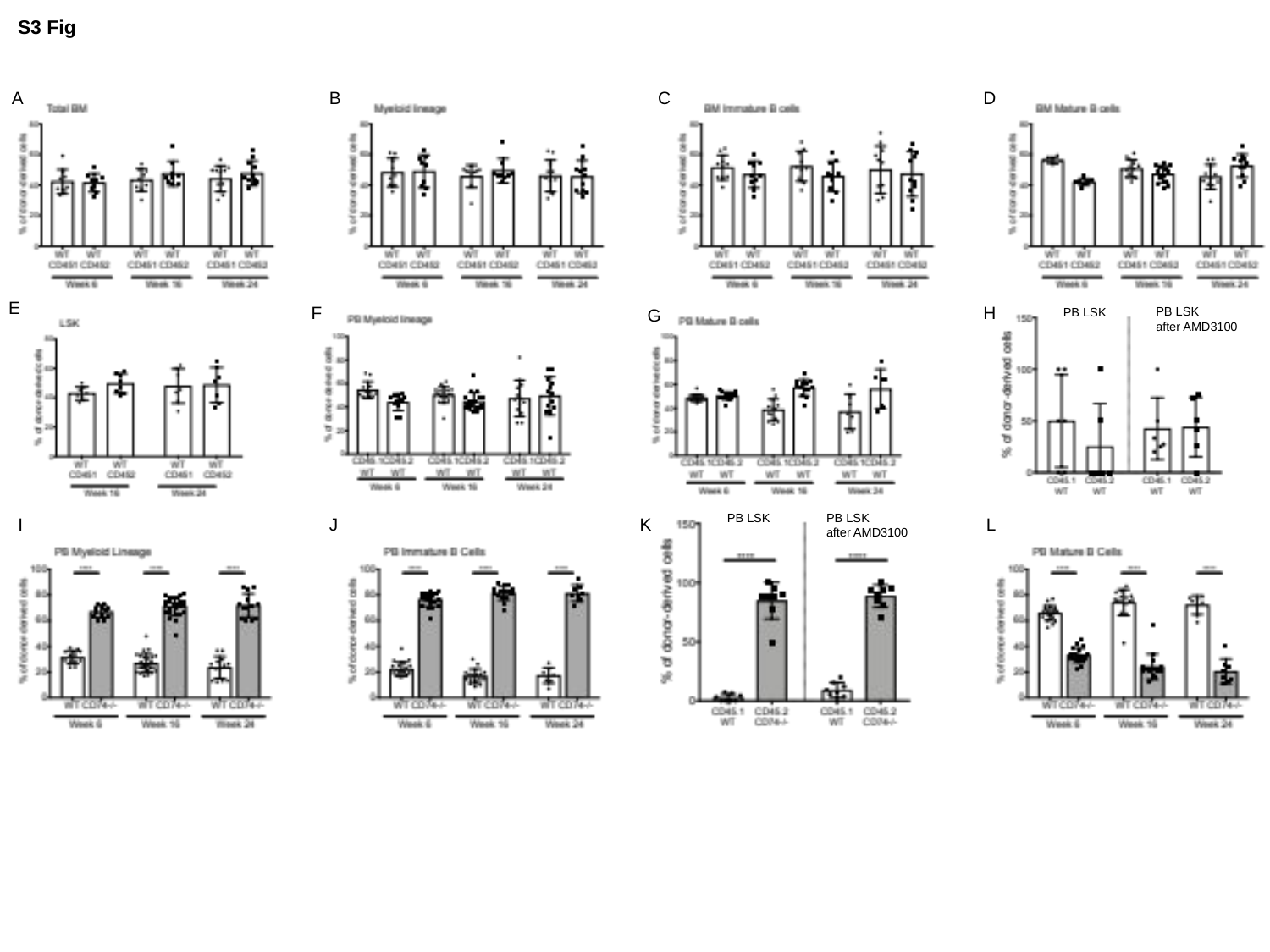

S3 Fig
A
B
C
D
E
F
H
G
PB LSK
after AMD3100
PB LSK
PB LSK
PB LSK
after AMD3100
I
J
K
L

Supplement: S3 Fig — (A–H) WT (CD45.1):WT (CD4.2) chimeric mice maintain a 1:1 ratio of donor cells. Lethally irradiated WT (CD45.1) mice were transplanted with BM derived from WT (CD45.2) mice at a 1:1 ratio. Percent of each population in the BM and PB was analyzed after 6, 16, and 24 weeks. (A) Total BM cells; Data A in S11 Data (B) BM myeloid cells; Data B in S11 Data (C) immature BM B cells; Data C in S11 Data (D) mature BM B cells; Data D in S11 Data (E) BM LSK; Data E in S11 Data (F) PB myeloid cells; Data F in S11 Data (G) PB mature B cells; Data G in S11 Data (H) PB LSK, and 4 h after injection of AMD3100; Data H in S11 Data (I–L) Lethally irradiated WT (CD45.1) mice were transplanted with BM derived from WT (CD45.1) and CD74−/− (CD45.2) mice at a 1:1 ratio. Percent of donor-derived cells was analyzed in the PB after 6, 16, and 24 weeks in (I) myeloid cells; Data I in S11 Data (J) immature BM B cells; Data J in S11 Data (K) PB LSK and 4 h after injection of AMD3100; Data K in S11 Data (L) PB mature B cells; Data L in S11 Data, n = 6–27. Bars show SEM. Unpaired two-tailed t test *p < 0.05; **p < 0.01; ***p < 0.001; ****p < 0.0001. BM, bone marrow; PB, peripheral blood; WT, wild-type. (PPTX) [file pbio.3001121.s003.pptx]

## Slide 1
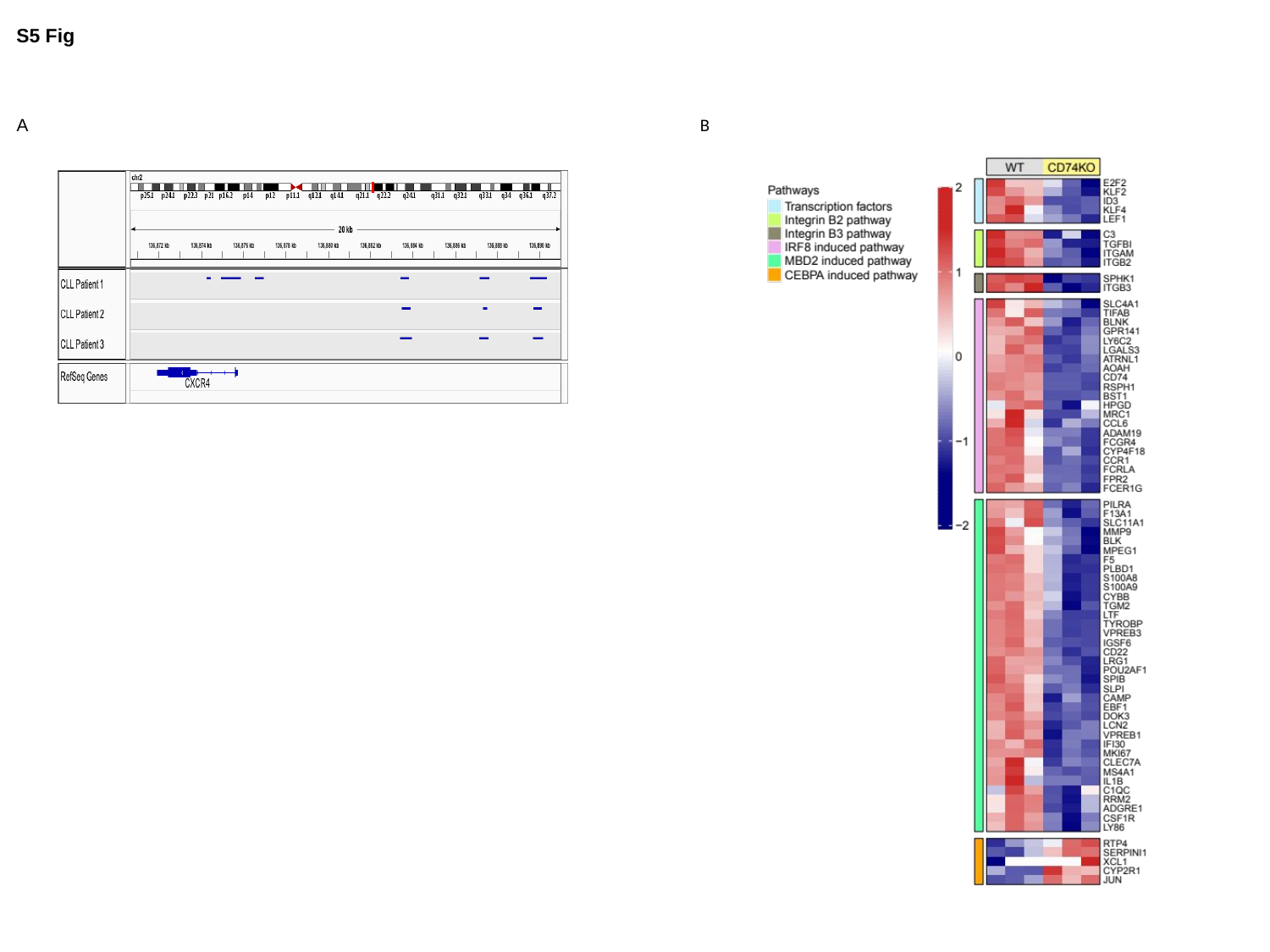

S5 Fig
A
B

Supplement: S5 Fig — (A) Binding of CD74–ICD to CXCR4 promoter region in CLL cell samples. ChIP-seq analysis using anti-CD74 antibody. (B) HSCs (CD34-/LSK) (103 cells) were sorted from WT and CD74−/− mice. Differentially expressed genes were identified using DESeq2 (version 1.10.1); n = 3. Analysis of the pathway was performed using Enrichr, a comprehensive gene set enrichment analysis. ChIP-seq analysis, chromatin immunoprecipitation-sequencing; HSC, hematopoietic stem cell; WT, wild-type. (PPTX) [file pbio.3001121.s005.pptx]

## Slide 1
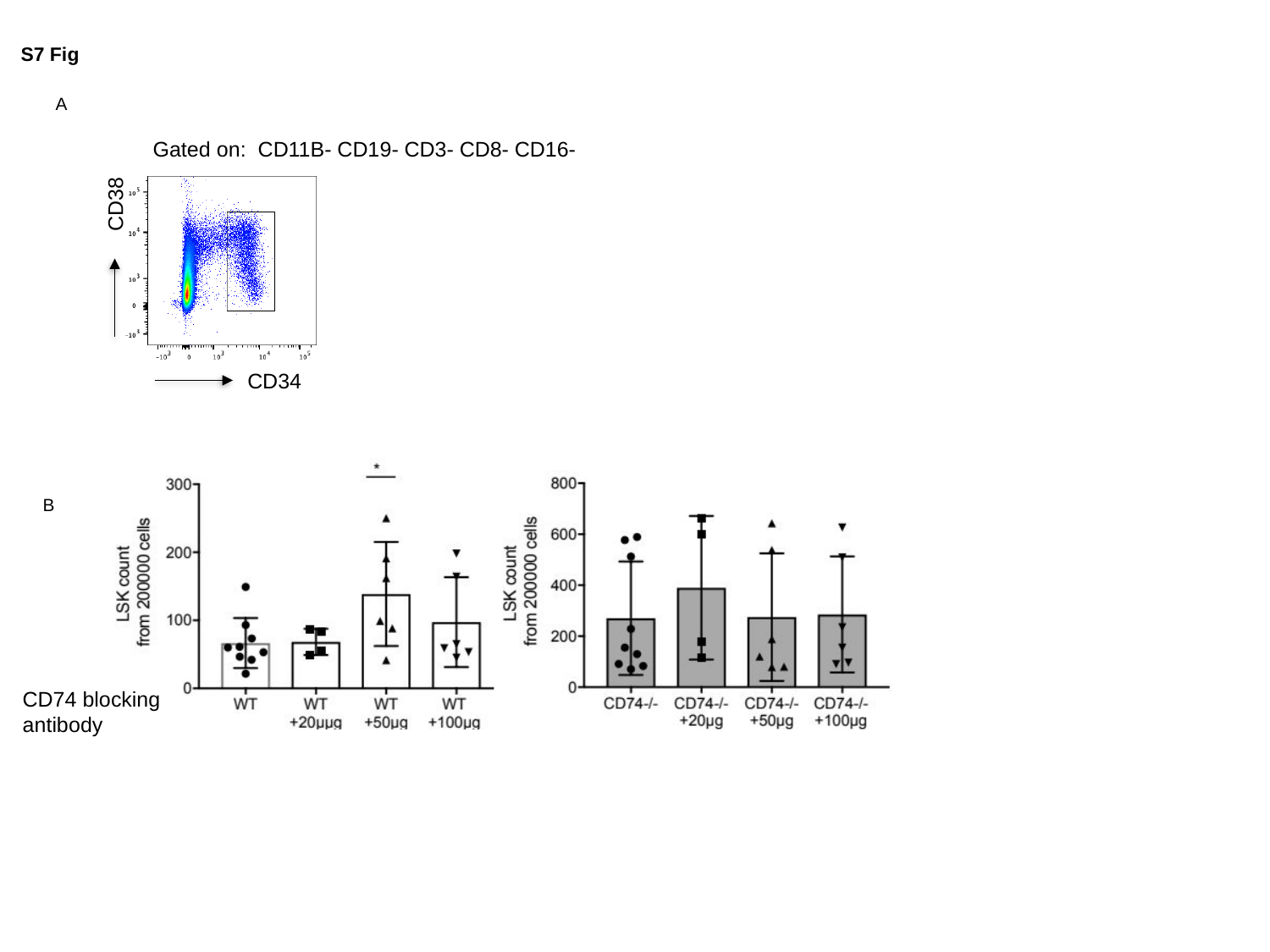

S7 Fig
A
Gated on: CD11B- CD19- CD3- CD8- CD16-
CD38
CD34
B
CD74 blocking
antibody

Supplement: S7 Fig — (A) Gating strategy for human CD34+ cells. (B) WT and CD74−/− BM cells were cultured alone or incubated with blocking anti-CD74 antibody (20, 50, and 100 mg/ml). After 48 h, LSK expressing cells from 200,000 cells were analyzed by FACS; n = 4–7, Data A in S14 Data. The underlying numerical data for this figure can be found in S14 Data, and fcs files and gates can be found in FR-FCM-Z3F2. BM, bone marrow; FACS, fluorescence-activated cell sorting; WT, wild-type. (PPTX) [file pbio.3001121.s007.pptx]
