# Supplementary material for: CD74 is a regulator of hematopoietic stem cell maintenance
Source: PLoS Biol. 2021 Mar 4;19(3):e3001121. doi: 10.1371/journal.pbio.3001121 (PMC7963458; doi:10.1371/journal.pbio.3001121)
Supplement: S4 Fig — WT and CD74−/− HSPCs home similarly to the BM. (A–E) Lethally irradiated CD45.1 recipient mice were reconstituted with 25*106 of either WT (CD45.2) or CD74−/− (CD45.2) BM cells. Percent of Lin- and HSPCs in the BM was evaluated at 24 h (A) Data A in S12 Data, 72 h (B, C) Data B and C in S12 Data and 1 week (D, E), Data D and E in S12 data, posttransplant; n = 4–7. (F) Lethally irradiated WT (CD45.1) mice were transplanted with BM derived from WT (CD45.1) and WT (CD45.2) at a 3:1 ratio, or BM derived from WT (CD45.1) and CD74−/− (CD45.2) mice at a 3:1 ratio. Mice were analyzed 16 weeks after transplantation. Dot plots show the chimerism in the BM at the end of the experiment. (G–L) Lethally irradiated WT (CD45.1) mice were transplanted with BM derived from WT (CD45.1) and WT (CD45.2) at a 7:1 ratio, or BM derived from WT (CD45.1) and CD74−/− (CD45.2) mice at a 7:1 ratio. Mice were analyzed 16 weeks after transplantation. Percent of each population in the BM was analyzed 13 weeks; n = 9–13. (G) Total BM cells; Data F in S12 Data (H) myeloid cells; Data G in S12 Data (I) immature BM B cells; Data H in S12 Data (J) LSK, Data I in S12 Data (K) CD34-/LSK, Data J in S12 Data (L) mature BM B cells, Data K in S12 Data. Bars show SEM. Unpaired two-tailed t test *<0.05 **<0.01 ***<0.001 ****<0.0001. The fcs files and gates can be found in FR-FCM-Z3F2. BM, bone marrow; HSPC, hematopoietic stem and progenitor cell; WT, wild-type. (PPTX) [file pbio.3001121.s004.pptx]

## Slide 1
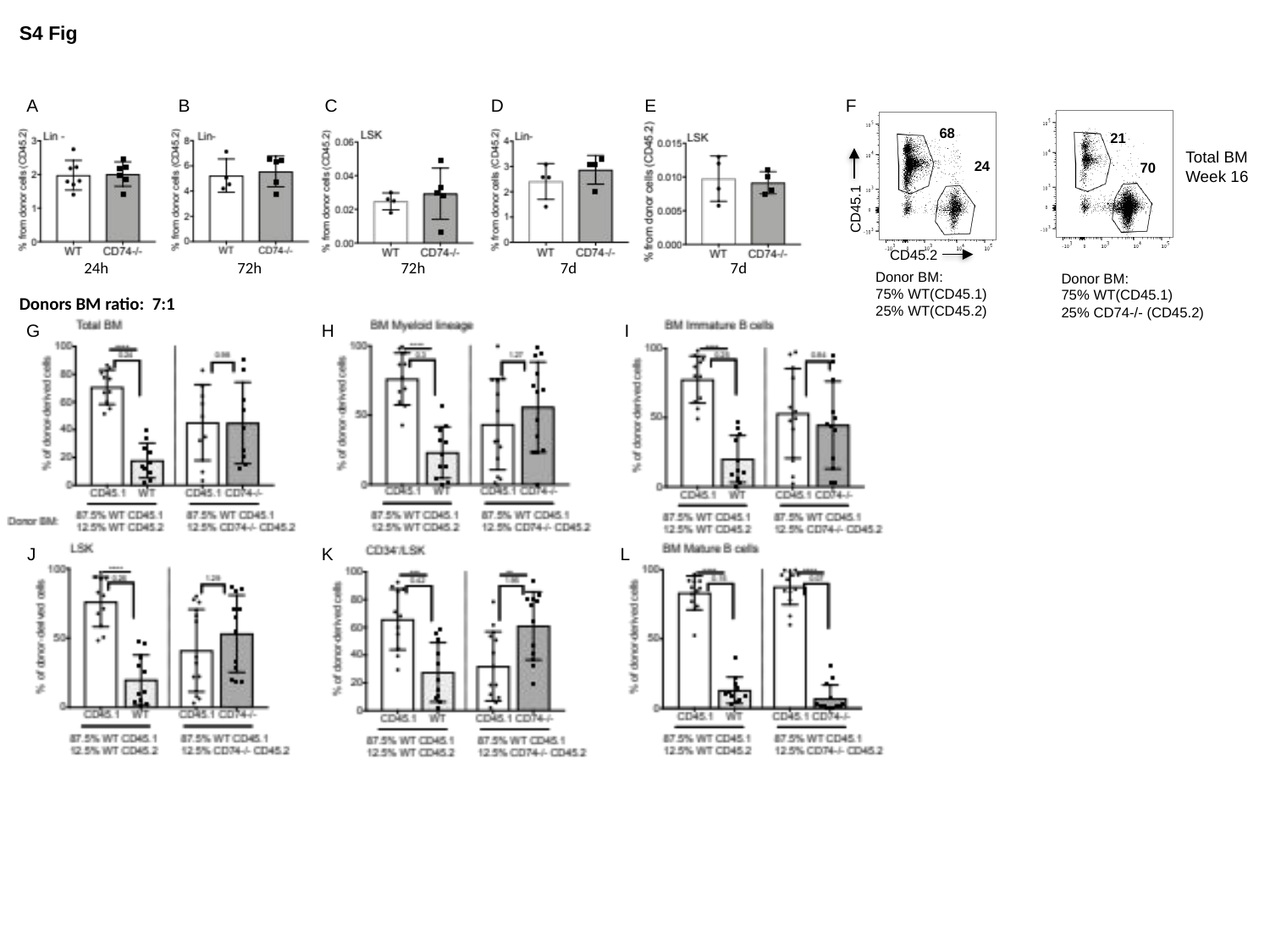

S4 Fig
A
B
C
D
E
F
21
70
CD45.2
Donor BM:
75% WT(CD45.1)
25% CD74-/- (CD45.2)
68
Total BM
Week 16
24
CD45.1
Donor BM:
75% WT(CD45.1)
25% WT(CD45.2)
24h
72h
72h
7d
7d
Donors BM ratio: 7:1
G
H
I
J
K
L
