# Supplementary material for: CD74 is a regulator of hematopoietic stem cell maintenance
Source: PLoS Biol. 2021 Mar 4;19(3):e3001121. doi: 10.1371/journal.pbio.3001121 (PMC7963458; doi:10.1371/journal.pbio.3001121)
Supplement: S6 Fig — (A) FACS analysis of LSK from WT and CD74−/− mice for CD18; n = 6, Data A in S13. (B) FACS analysis of LSK from WT and MIF−/− mice for CD18; n = 3, Data B in S13 Data. (C) WT and CD74−/− BM were cultured with MIF inhibitor (ISO-1) for 48 h, and percent CD18 on LSK was analyzed; n = 6, Data C in S13 Data (D) WT (CD45.1) Lin negative cells were cultured in the presence of WT (CD45.2) total BM or MIF−/− (CD45.2) total BM for 48 h, the percent CD18 on LSK cells (CD45.1) was analyzed by FACS; n = 8, Data D in S13 Data (E) LSK and CD34-/LSK populations from WT and CD74−/− mice were analyzed for cell surface expression of CD11A, CD11B, and CD11C; n = 6 by FACS. Data E in S13 Data. (F) WT and CD74−/− BM were cultured with the MIF inhibitor (ISO-1) for 48 h, and percent CD11B on LSK and CD34-/LSK was analyzed by FACS; n = 4, Data F in S13 Data. Bars show SEM. Unpaired two-tailed t test *p < 0.05; **p < 0.01; ***p < 0.001; ****p < 0.0001. The fcs files and gates can be found in FR-FCM-Z3F2. BM, bone marrow; FACS, fluorescence-activated cell sorting; MIF, migration inhibitory factor; WT, wild-type. (PPTX) [file pbio.3001121.s006.pptx]

## Slide 1
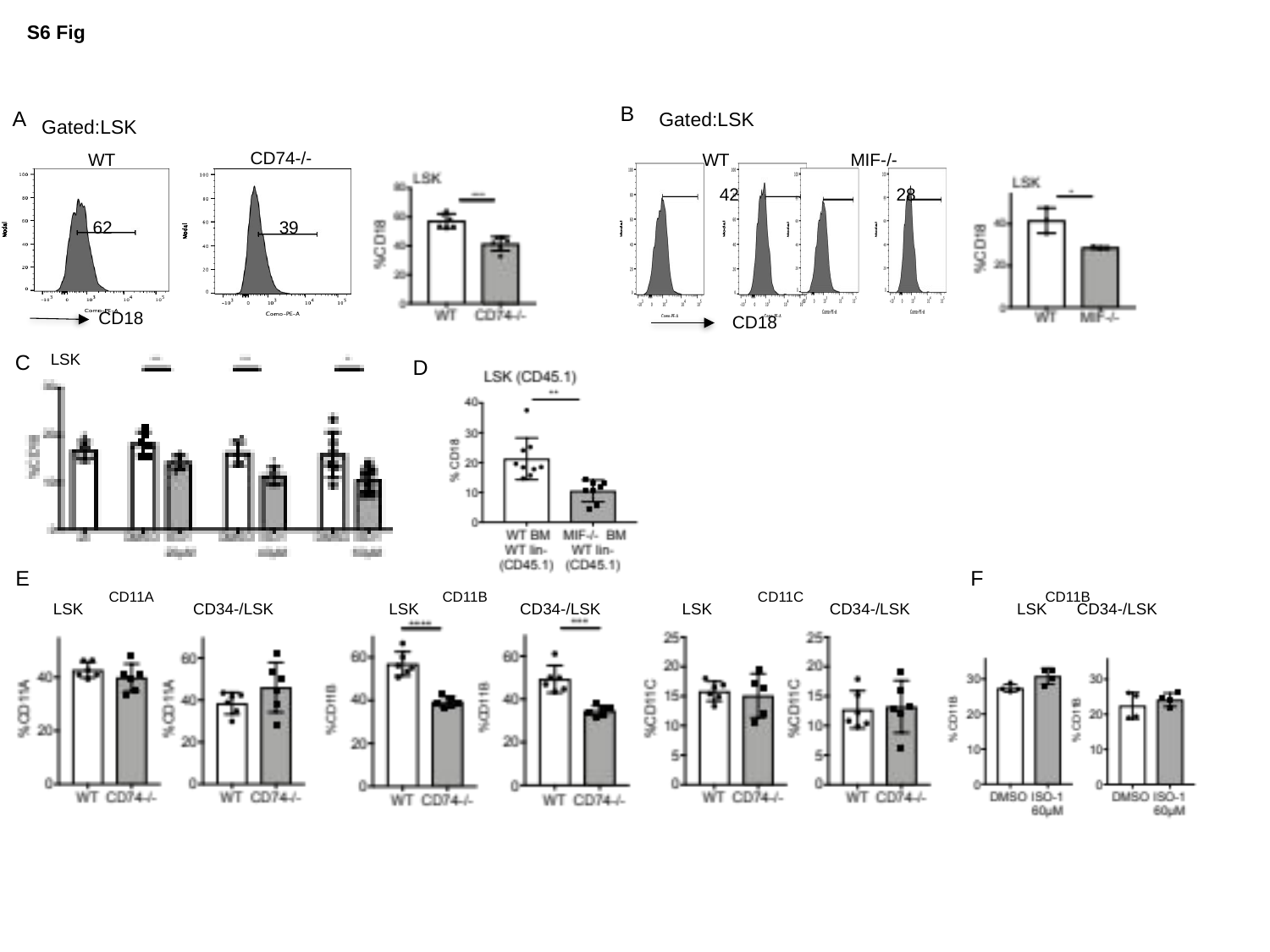

S6 Fig
B
Gated:LSK
WT
MIF-/-
42
28
CD18
A
Gated:LSK
CD74-/-
WT
62
39
CD18
C
LSK
D
E
F
CD11A
CD11B
CD11C
CD11B
LSK
CD34-/LSK
LSK
CD34-/LSK
LSK
CD34-/LSK
LSK
CD34-/LSK
