# Supplementary material for: CD74 is a regulator of hematopoietic stem cell maintenance
Source: PLoS Biol. 2021 Mar 4;19(3):e3001121. doi: 10.1371/journal.pbio.3001121 (PMC7963458; doi:10.1371/journal.pbio.3001121)
Supplement: Attachment — Submitted filename: Letter revision NOV 16.docx [file pbio.3001121.s022.docx]

November 16^th^, 2020

Dear Editor,

Thank you for your letter of May 1^st^ concerning our manuscript “***CD74 is a regulator of hematopoietic stem cell maintenance***”. We are gratified that the reviewers found substantial merit in our study. Nevertheless, the referees raised issues that they felt precluded publishing our manuscript in its present form; we have now addressed these issues in our revised manuscript, as described below:

*Reviewer 1*

1. *The main novelty of this paper is the finding that the CD74 deficiency results in expansion of the highly potent HSC population. Regarding the molecular mechanism, this paper has focused on retention of HSCs and HSPCs. It would be very nice, if they could see how CD74 molecules works for HSC retention with CD18.*

Our results demonstrate that the accumulation of CD74 deficient hematopoietic stem cells in the BM results from their increased potential to repopulate and compete for BM niches rather than increased retention in this compartment. We show that CD74 regulates the maintenance of HSPCs, and CD18 expression. Although CD18 is part of the integrin complex together with an α subunit, and can regulate cell adhesion and retention, reduced levels of CD18 in HSC in mouse showed expansion of HSCs in the bone marrow [1] and in human downregulation of CD18 expression was associated with a primitive HSC phenotype and enhanced long-term repopulating capacity in NSG mice [2]. Therefore, although we also show some effect on CXCR4 expression and cell retention, the main abnormality demonstrated in cells lacking CD74 is in their induced survival and cell maintenance.

To provide more insight into the mechanism of action of CD74, we further analyzed the regulation of CD18 by the CD74 ligand, MIF, by blocking its activity with the inhibitor, ISO-1. As shown in the new Fig 7N and new Supplementary Fig 6C, blocking MIF reduced CD18 cell surface expression on LSK and CD34 negative cells.

To further follow the role of extracellular MIF in the maintenance of HSPCs and CD18 expression, WT HSPCs were co-cultured with WT or MIF-/- BM cells. As shown in the new Fig 7O, and new Supplementary 6D, MIF secreted from the BM cells was essential for CD18 cell surface expression. Therefore, we suggest that MIF secreted from HSPCs or the BM environment binds to CD74 and induces CD18 expression, and in the absence of MIF or CD74, its expression is downregulated.

Next, we analyzed the complexes formed by CD18 in HSPCs. Integrins are heterodimeric adhesion molecules that mediate cell interactions with the extracellular matrix. β_2_ integrins are composed by the β_2_ subunit (CD18) and variable α subunits (CD11). The expression of β_2_ integrins is restricted to the hematopoietic system and includes four members, whose identity depends on the identity of the α subunit: CD18:CD11A (LFA-1), CD18:CD11B (Mac-1), CD18:CD11C (p150.95), and CD18:CD11D (α_D_β_2_) are associated with distinct patterns of expression and distinct functions in the hematopoietic system [3, 4]. In mouse models, the absence of CD11A^−^ HSCs resulted in higher engraftment as compared to similar numbers of CD11A^+^ HSCs, suggesting that the reduced expression of CD11A was associated with a more primitive HSC phenotype [5]. Similarly, in nonhuman primates, the CD34^+^CD11B^−^ fraction of HSCs are capable of repopulating both lymphoid and myeloid lineages [6].

To identify the β2 integrin complex regulated by CD74, we first analyzed the α subunits that are affected by CD74 deficiency. As shown in the new Supplementary Fig 6E, CD74-/- HSPCS express lower levels of CD11B, but not of CD11A or CD11C. The RNA-seq results (Supplementary Fig 5B) support this finding (shown in the heatmap CD11B/ ITGAM). However, blocking CD74 activation by ISO-1 did not affect CD11B cell surface levels (new Supplementary Fig 6F), suggesting that CD74 does not directly regulate CD11B expression.

2. *According to the result of the effect of CD74 blockade and the role of CD74 in haematological recovery from chemotherapy with 5-FU treatment, it helps improve clinical insight into bone marrow transplant protocols and enable improved engraftment. You should discuss about the more practical translation in the discussion section.*

We added several sentences to the Discussion section (final paragraph) to provide more insight into the practical aspects of clinical translation of CD74 inhibition.

3. *The experiments are well-designed, while it may be more convincible if comprehensive analysis is explored. Such as, BM populations of the mixed chimeras at various time points are showed in both WT CD45.2 VS. WT CD45.1 and CD74-/- (CD45.2) VS. WT CD45.1. How about PB populations of the mixed chimeras at various time in WT CD45.2 VS. WT CD45.1 and the total PB and LSK in CD74-/- (CD45.2) VS. WT CD45.1? (Figure 3 and Supplementary Figure 3.)*

We added to the manuscript new Supplementary Fig 3 F-H showing the PB populations in WT CD45.2 versus WT CD45.1 mixed chimera. We further added the new Supplementary Fig 3 K showing an elevation of the CD74 deficient LSK population in the PB of the chimeric mice in the presence or absence of the CXCR4 inhibitor, AMD3100. Supplementary Fig 3 H shows LSK populations in PB in WT CD45.2 versus WT CD45.1 mixed chimera.

*And also, what's the additional findings, like survival-related pathways or other genes except CD18, are affected by CD74-/- in your RNA-seq data? (Figure 7-J)*

Additional analysis of the RNA-seq results demonstrates that besides regulation of CD18 expression, CD74 is involved in the control of various pathways that are known to regulate HSCP maintenance (new Supplementary Fig 5B).

4. *Most conclusions are well-supported by the data and well-designed experiment. However, there are few conclusions maybe need further discuss. For example, In Figure 2 A-C, the ratio of % from total BM cells in LIN-, LSK, and CD34-/LSK subpopulation between CD74-/- BM and WT donors is around 1-3. In Figure 2 E-F, the ratio % LSK from total BM between CD74-/- and WT stem cell numbers under cellular microenvironment (stroma, cytokines (TPO and SCF)) is also around 1-3*

The results presented in Fig 2 A-C and Fig 2 E-F are not related. Fig 2 A-C demonstrates the role of the in vivo microenvironment in chimeric mice. In Fig 2 E-F, WT or CD74-/- BM cells were cultured ex vivo with the support of stroma or cytokines for 48h. Both experiments show that the absence of CD74 expression by the stem cells themselves leads to their accumulation. However, the conditions in the microenvironment had only a minor or no effect on the fold of increase in WT and CD74^-/-^ stem cell numbers, further demonstrating that the accumulation potential of HSPCs in the absence of CD74 is intrinsic to the cells.

5. *They have mentioned "These conditions (cellular microenvironment) had only a minor or no effect on the fold of increase in WT and CD74-/- stem cell numbers" and draw the conclusion "the accumulation potential of HSPCs in the absence of CD74 is intrinsic to the cells."
   How about the fold change in LIN- and CD34-/LSK between CD74-/- and WT? It may be more clear if they show the fold change between CD74-/- and WT within the same condition and put the fold change number in Figure 2 A-C as well, and also in Figure 5.*

We add fold change to Fig 2 A-C and Fig 5 A-L.

6. *When check the H-K, in the WT:CD74-/- chimera, the numbers of BM Myeloid, immature, LSK and CD34-/LSK is different, especially CD34-/LSK. It is difficult to be convinced when describe them "equal" in the paper. How about adjust to 5:1 dilution or 9:1 and then check the difference?*

To address the reviewer’s comment, we generated a 9:1 chimera (Figure 5 G-L) and followed the various populations in the BM and periphery (The 7:1 chimera is now in Supplementary Fig 4 G-L).

While in the WT versus CD74-/- 3:1 ratio chimera, the CD74 deficient cells display a growth advantage in the different populations, this advantage diminished in the 9:1 ratio chimera. However, even at a 9:1 ratio, the ratio of CD74-/- cells (CD45.2) to WT (CD45.1) was higher than the ratio of WT CD45.2 to WT CD45.1. Thus, CD74-deficient HSCs have a stronger potential to repopulate the BM and compete for the niches relative to WT HSPCs.

7. *In their previous paper (Gore Y et al, J Biol Chem, 2008), it shows that MIF binds to a complex of CD74 and CD44, resulting in initiation of a signaling pathway. They should discuss about the role of CD44 and the change of the signaling cascade.*

To analyze the role of CD44 in the maintenance of HSPCs, we determined its expression in these cells. As shown in the new Supplementary Fig 1 J-K, no difference in CD44 mRNA (Supplementary Fig 1J) and protein levels (Supplementary Fig 1K) was detected in WT versus CD74 deficient cells. These results imply that CD44 does not play a role in the regulation induced by CD74. Also, our results show that the hypoxic microenvironment contributes to the CD74-induced outcome, providing an advantage for the CD74-deficient HSPCs. Thus, CD74 induces a cell type-specific cascade that leads to different cell death outcomes.

Minor comments:

1. *Figure 1-B, -I, -J, -K, Figure 2-C, Figure 5-G, -H, -I, -J, -K, Figure 6-D, -E, G, Figure 7-A, -B, Figure 8-A and also Supplementary Figure 4-C and -E, the error bars are relatively high. May I ask what's your interpretation?*

In each experiment we analyzed a large number of mice. The error bars represent the different staining intensities between experiments.

2. *Spelling mistakes. e.g. 'may be' (Page 6), 'TPO' (instead 'EPO') (Page 24, line 2), 'WT (CD45.2)' (Supplementary Figure 3), 'WT (CD45.1)' (Figure 3-A).*

TPO is an abbreviation for Thrombopietin. Spelling mistakes were corrected.

*3) The format. e.g. '2Internal' (Page 8, line 5), the sentence should be bold (Page 12, line 1 and Supplementary Figure 5), divide the subtitle and the paragraph (Page 23, line 18 and line 24), the arrow in the figure. (Figure 1- A and D, Supplementary Figure 1 A-D, Figure 3, Figure 6, Figure 7, Supplementary Figure 4 and Supplementary Figure 6.)*

Revised

4*) The font. e.g. Lin- VS. Lin-, CD34+ VS. CD34+, CD34- VS. CD34-, Gated: LIN- VS. Gated:Lin-, CD74-/- VS. CD74-/-, MIF-/- VS. MIF-/-, Sca-1+ VS. Sca1+. (Figure 1, Supplementary Figure 1, Supplementary Figure 2, Page 12, line 3, line 13-14 and line 21.)*

Revised

5) *Without Space. e.g. '% from' (Figure 1-I), 'per 15000' (Figure 1-K), '25% WT' (Supplementary Figure 4-F).*

Revised

6) *Others. e.g. capital (Supplementary Figure 3), '0.25 x 106' (Page 23, line 21), grammar mistake (Page 17, line 23), etc.*

Revised

*Reviewer 2:*

*Major comments:*

8. *The authors convincingly demonstrate that the CD74 deletion in non-hematopoietic cells does not contribute to the phenotype, but the experiments do not exclude the possibility that CD74 in other hematopoietic cells plays a role. This would fit with the kinetics since the effects take some time to manifest after transplantation - perhaps should be discussed.*

In Fig 2 A-C we analyzed chimeric mice with different microenvironments (non-hematopoietic cells from WT or CD74-/-) and we showed that the phenotype of the donor cells and not that of the microenvironment is responsible for the expansion of CD74-/- HSC. However, as the reviewer commented, we cannot exclude the possibility that other immune cell populations are responsible for the phenotype of CD74-/- HSPC expansion. We now note this issue in the Discussion section.

1. *The effects of CD74 are clearly cell-autonomous but still require MIF binding? A number of growth factors was used but was MIF not used for in vitro culture? Is MIF produced by HSCs and acting through an autocrine loop?*

To follow the role of MIF in the maintenance of HSPCs, its intracellular levels in these cells was analyzed. As shown in the new Supplementary Fig 1 H-I, similar levels of MIF mRNA (new Supplementary Fig 1H) and protein levels of this cytokine (Supplementary Fig 1I) were detected in CD74 deficient and WT HSCPs. We further analyzed the role of MIF in CD18 expression. Our results show that CD18 expression is regulated by CD74 on HSPCs (Fig 7 J-M). In addition, as shown in the new Fig 7N and new Supplementary Fig 6C, blocking MIF reduced CD18 cell surface expression on LSK and CD34 negative cells. To further follow the role of extracellular MIF in the maintenance of HSPCS and CD18 expression, WT HSPCs were co-cultured with WT or MIF-/- BM cells. As shown in the new Fig 7O, and Supplementary Fig 6D, MIF secreted from the BM cells was essential for CD18 cell surface expression. In its absence, CD18 expression was reduced. Therefore, we suggest that MIF secreted from HSPCs or the BM environment binds to CD74 and induces CD18 expression, and in the absence of MIF or CD74, its expression is downregulated.

2. *Please explain how you define the different cell populations (including mature and immature B cells) immunophenotypically in the figure legend.*

We explained the gating strategy in the legend to Figure 3, in Supplementary Fig 1 A-D , and new Supplementary 7A.

3. *The authors propose several potential mechanisms explaining the increased HSC survival in CD74 KO but no definitive functional explanation. Different survival effects on HSCs and B cells is very intriguing. The authors hypothesize that increased hypoxia in HSCs might explain the difference and look into Hif1a. If CD74 induces Hif1a transcription, why are CD74 KO cells more responsive and fit under hypoxia, which stabilizes Hif1a?*

The reviewer raised an important point that needs to be further discussed. It was shown in several studies that downregulation of HIF-1α expression inhibits precursor cells differentiation [7, 8]. We therefore suggest that the lower levels of HiF1α enable the accumulation of more primitive cells. We address this point in the Discussion section.

4. *Since the last time point in Fig. 8C is very close to the point where the curves separate from each other it would be best to show longer evolution.*

The experiment in Fig 8C was repeated with a lower concentration of 5-FU (125 mg/kg), which allowed us to prolong the experiment. As shown in the new Fig 8D, CD74 deficient mice injected with the lower concentration of 5-FU survived longer than WT mice.

5. *Have the authors tested human HSPCs (like cord-blood-derived, which typically show reduced engraftment) with the CD74 blocking antibody? Not essential but would substantiate the human relevance.*

To address this comment, we added to the manuscript an experiment using human HSPCs (Gating strategy appears in the new Supplementary Fig 7A). CD74 staining on human CD34+ is shown in the new Fig 8E. Blocking CD74 expressed on these cells resulted in downregulation of CD18 expression (New Fig 8F). The role of CD18 was shown in human HSCs; downregulation of CD18 expression was associated with a primitive HSC phenotype and enhanced long-term repopulating ability in NSG [2]. These results suggest that CD74 regulates a similar pathway in human HSPCs.

6. *They show the frequency of LSK cells in BM cells treated in vitro with anti-CD74 Ab, but this could reflect effects on other cell populations - enumerating LSK cells would be better.*

We added cell numbers in the new Supplementary Fig 7B.

7. *Could the blocking antibody be well tolerated in vivo or cause lymphopenia?*

We suggest using this antibody ex-vivo, and then transplanting patients with the CD74 treated culture. In this way the side effects of CD74 blocking will be limited and the effect will be only on the transplanted population.

Minor comments:

P*.6 1st para. Rephrase concluding remark to "MIF/CD74 limits HSPC numbers, as opposed to the prosurvival effect in B cells" or similar.*

We rephrased the concluding remark

P*. 6. 2nd para. Refine "Thus, the lack of CD74 in hematopoietic cells…"
T cells last longer but should be eventually replaced by donor-derived cells.*

We tried to ablate the host T cells using a different protocol of irradiation (450rad, and after 3 days, 950rad), but we still detected between 30-50 % T cells from the host even after 4 months.

*For the CXCR4 part, there seem to be two antagonistic changes which might explain unchanged mobilization by AMD3100 despite lower CXCR4 expression (higher number of BM HSPCs but lower CXCR4 expression) - perhaps deserves some comment in the discussion.*

A comment regarding these effects was added to the text

We believe that the results presented and clarifications described here strengthen this study and further substantiate our conclusions. We would therefore appreciate your continued consideration of this manuscript for publication in PLOS Biology.

Sincerely,

Idit Shachar

***References***

1.	Leon-Rico D, Aldea M, Sanchez R, Segovia JC, Weiss LA, Hidalgo A, et al. Brief report: reduced expression of CD18 leads to the in vivo expansion of hematopoietic stem cells in mouse bone marrow. Stem Cells. 2014;32(10):2794-8. Epub 2014/06/07. doi: 10.1002/stem.1762. PubMed PMID: 24906078.

2.	Mesa-Nunez C, Leon-Rico D, Aldea M, Damian C, Sanchez-Baltasar R, Sanchez R, et al. The downregulated membrane expression of CD18 in CD34(+) cells defines a primitive population of human hematopoietic stem cells. Stem Cell Res Ther. 2020;11(1):164. Epub 2020/04/30. doi: 10.1186/s13287-020-01672-0. PubMed PMID: 32345365; PubMed Central PMCID: PMCPMC7189462.

3.	Hynes RO. Integrins: bidirectional, allosteric signaling machines. Cell. 2002;110(6):673-87. PubMed PMID: 12297042.

4.	Tan SM. The leucocyte beta2 (CD18) integrins: the structure, functional regulation and signalling properties. Biosci Rep. 2012;32(3):241-69. Epub 2012/03/31. doi: 10.1042/BSR20110101. PubMed PMID: 22458844.

5.	Fathman JW, Fernhoff NB, Seita J, Chao C, Scarfone VM, Weissman IL, et al. Upregulation of CD11A on hematopoietic stem cells denotes the loss of long-term reconstitution potential. Stem Cell Reports. 2014;3(5):707-15. Epub 2014/11/25. doi: 10.1016/j.stemcr.2014.09.007. PubMed PMID: 25418718; PubMed Central PMCID: PMCPMC4235136.

6.	Van Beusechem VW, Bart-Baumeister JA, Bakx TA, Kaptein LC, Levinsky RJ, Valerio D. Gene transfer into nonhuman primate CD34+CD11b- bone marrow progenitor cells capable of repopulating lymphoid and myeloid lineages. Hum Gene Ther. 1994;5(3):295-305. Epub 1994/03/01. doi: 10.1089/hum.1994.5.3-295. PubMed PMID: 7517188.

7.	Choo SY, Yoon SH, Lee DJ, Lee SH, Li K, Koo IH, et al. Runx3 inhibits endothelial progenitor cell differentiation and function via suppression of HIF-1alpha activity. Int J Oncol. 2019;54(4):1327-36. Epub 2019/04/11. doi: 10.3892/ijo.2019.4713. PubMed PMID: 30968151.

8.	Ng KM, Lee YK, Chan YC, Lai WH, Fung ML, Li RA, et al. Exogenous expression of HIF-1 alpha promotes cardiac differentiation of embryonic stem cells. J Mol Cell Cardiol. 2010;48(6):1129-37. Epub 2010/02/02. doi: 10.1016/j.yjmcc.2010.01.015. PubMed PMID: 20116384.
