# Supplementary material for: CD74 is a regulator of hematopoietic stem cell maintenance
Source: PLoS Biol. 2021 Mar 4;19(3):e3001121. doi: 10.1371/journal.pbio.3001121 (PMC7963458; doi:10.1371/journal.pbio.3001121)
Supplement: Attachment — Submitted filename: letterhead full professor.docx [file pbio.3001121.s023.docx]

December 28^th^, 2020

Dear Dr. Hodge,

Thank you for your letter of December 17^th^ concerning our manuscript “***CD74 is a regulator of hematopoietic stem cell maintenance***”. We are gratified that the reviewers found substantial merit in our study. Nevertheless, there were issues that precluded publishing our manuscript in its present form; we have now addressed these issues in our revised manuscript, as described below:

ETHICS STATEMENT:

We ask that you please include the following details:

(a)	Please amend your current ethics statement to include the full name of the institutional review boards that approved your specific study, including the names of the specific hospitals. We additionally ask that you include your IRB approval numbers in your ethics statement.

**Human BM cells derived from healthy patients were provided by the hematology institute of the Kaplan Medical Center in compliance with the review board of the hospital. IRB number 1339-1.**

**The information was included in the Cells section in Materials and Methods section.**

(b)	Please provide additional details regarding participant consent for the use of bone marrow cells from healthy patients. In the ethics statement in the Methods and online submission information, please ensure that you have specified (1) whether consent was informed and (2) what type you obtained (for instance, written or verbal, and if verbal, how it was documented and witnessed). If your study included minors, state whether you obtained consent from parents or guardians. If the need for consent was waived by the ethics committee, please include this information. On the other hand, if the patient samples were accessed anonymously, please include this information.

**The samples were obtained by written consent.  The information was included in the text.**

(c)	Please include the approval number issued by the Weizmann IACUC for the animal studies conducted in your manuscript.

**IACUC numbers:2016-2019 - 27730616-2 and for 2019-2022 - 15340619-2. The information was included in the text.**

DATA:

Supplementary files (e.g., excel). Please ensure that all data files are uploaded as 'Supporting Information' and are invariably referred to (in the manuscript, figure legends, and the Description field when uploading your files) using the following format verbatim: S1 Data, S2 Data, etc. Multiple panels of a single or even several figures can be included as multiple sheets in one excel file that is saved using exactly the following convention: S1_Data.xlsx (using an underscore).

Regardless of the method selected, please ensure that you provide the individual numerical values that underlie the summary data displayed in the following figure panels as they are essential for readers to assess your analysis and to reproduce it:

Fig. 1B, C, E-K; Fig. 2A-F; Fig. 3B-H; Fig. 4A-E; Fig. 5A-P; Fig. 6B, D-K; Fig. 7A-G, I, J, L-O; Fig. 8A-F; Fig. S1E-K; Fig. S2A-D; Fig. S3A-L; Fig. S4A-E, G-L; Fig. S6A-F and Fig. S7B

**Individual numerical values were provided as data files and were uploaded as supporting information.**

For figures containing FACS data, we ask that you provide FCS files and a picture showing the successive plots and gates that were applied to the FCS files to generate the figure.
**FACS data that contain plots and gates were uploaded and in the data review URL.**

*Please also ensure that figure legends in your manuscript include information on WHERE THE UNDERLYING DATA CAN BE FOUND where the underlying data can be found, and ensure your supplemental data file/s has a legend*

**The information where the data can be found was added to the legends.**

Finally, the RNA-Sequencing data presented in the manuscript should be deposited in a publicly available database such as GEO. We ask that you please amend your Data Availability Statement to list the name of the repository where the RNA-Seq can be found and the accompanying accession numbers. Please see <https://journals.plos.org/plosbiology/s/data-availability> for additional information.

**The RNA-Seq data discussed in this publication have been deposited in NCBI's Gene Expression Omnibus and are accessible through GEO Series accession number GSE163661 (https://www.ncbi.nlm.nih.gov/geo/query/acc.cgi?acc= GSE163661)."**

We would appreciate your input and your continued consideration of this manuscript for publication in PLOS Biology.

Sincerely,

Idit Shachar
